# Mapping evidence on the use of health promotion and disease prevention interventions as a strategy to sustaining pro-poor health insurance schemes: a scoping review protocol

**DOI:** 10.1186/s13643-022-01942-3

**Published:** 2022-04-15

**Authors:** Loretta Inkoom, Monica Ansu-Mensah, Vitalis Bawontuo, Desmond Kuupiel

**Affiliations:** 1 Faculty of Health and Allied Sciences, Catholic University College of Ghana, Fiapre, Ghana; 2 Department of Public Health Medicine, School of Nursing and Public Health, University of KwaZulu-Natal, Durban, 4001 South Africa; 3 Department of Health Services Management and Administration, School of Business, SD Dombo University of Business and Integrated Development Studies (SDD-UBIDS), Wa, Ghana; 4 Department of Global and International Health, School of Public Health, University of Development Studies (UDS), Tamale, Ghana; 5 Centre for Evidence-based Health Care, Division of Epidemiology and Biostatistics, Department of Global Health, Faculty of Medicine and Health Sciences, Stellenbosch University, Cape Town, 7530 South Africa

**Keywords:** Strategies, Sustainability, Health insurance, Healthcare, Health promotion, Preventive healthcare, Disease prevention, Public health, Pro-poor healthcare financing

## Abstract

**Background:**

Removing financial barriers and making healthcare accessible to all who need it remains an essential component of the United Nations’ sustainable development goals. Pro-poor healthcare financing schemes are policies that enable patients to concentrate on obtaining absolute medical care when needed rather than worrying about the cost of care. The demand for health services in healthcare facilities has increased tremendously due to the increasing burden of communicable and non-communicable diseases. This potentially threatens the sustainability of pro-poor health financing schemes. This study seeks to synthesize literature and map evidence on the use of health promotion and disease prevention interventions as a strategy to sustaining pro-poor health financing schemes globally.

**Methods:**

We will conduct a systematic scoping review utilizing the Arksey and O’Malley framework, Levac et al. recommendations, and the Joanna Briggs Institute guidelines. A comprehensive keyword search for relevant published articles will be conducted in MEDLINE through PubMed, Web of Science, Google Scholar, SCOPUS, CINAHL, and Science Direct from 1 January 2000 to the last search date in 2021. Limiters such as date and language (English) will be applied, but study design limitations will be removed during the search. Boolean term AND/OR Medical Subject Heading terms will also be included. The reference list of all included articles will also be searched for potentially eligible articles. Two investigators will independently screen the articles in parallel at the abstract and full-text stages using the eligibility criteria designed in a Google form. Charting of data will also be conducted independently by two investigators using a piloted data abstraction form and thematic analysis conducted. The emerging themes will be collated, summarized, and the results reported.

**Discussion:**

We hope to provide evidence of diverse health promotion and disease prevention policies/strategies used by countries to sustain their pro-poor health financing schemes for possible adoption by other countries. We also anticipate finding research gaps for further studies to help find innovative contextualized health prevention and promotion strategies to sustain pro-poor health financing schemes especially those in LMICs. The findings will be comprehensively discussed and disseminated at conferences and publication in a peer-reviewed journal.

**Supplementary Information:**

The online version contains supplementary material available at 10.1186/s13643-022-01942-3.

## Background 

Pro-poor healthcare financing schemes are policies that enable patients to concentrate on obtaining absolute medical care when needed rather than worrying about the cost of care [1]. Pro-poor healthcare financing policies such as the national health insurance scheme and free maternal healthcare are tailored towards improving access to healthcare and the provision of safe, quality, and effective healthcare to patients without financial hardship [2]. Most of these pro-poor healthcare financing policies were implemented during the pre-SDG era by some countries in their quest to make healthcare accessible as well as achieve the health-related Millennium Development Goals [1]. Although these pro-poor healthcare financing policies are yielding the desired results, evidence shows that they are bedeviled with many challenges, particularly in low-and-middle-come countries (LMICs) [3]. The demand for health services at healthcare facilities has increased tremendously. However, evidence shows that pro-poor healthcare financiers delay reimbursement of accrued cost from health services rendered to subscribers in health facilities particularly in some LMICs [4] which may threaten the sustainability of pro-poor health financing schemes in those countries.

Nonetheless, removing financial barriers and making health accessible to all who need it remains an essential component of the United Nations’ sustainable development goals (SDGs) [5]. To this end, it is imperative to sustain pro-poor health insurance schemes in order to continue to shield people from the financial risks of diseases who, otherwise, would not be able to finance their healthcare services [6, 7]. Sustainability of pro-poor health insurance schemes is also essential to maintain or improve upon the gains made following their implementation such as improved healthcare accessibility, reduction of maternal mortality, and reduced under-five mortality among others [8, 9]. Moreover, implementing pro-poor health insurance and the sustainability of health financing systems is fundamental for attaining Universal Health Coverage worldwide [10–13]. Hence, the sustainability of pro-poor health insurance schemes is vital in the face of an increasing incidence of preventable communicable and non-communicable diseases, a persistent increase in population growth rate, a rising cost of healthcare due to new technologies usage, and epidemiological shift [3].

The world is currently witnessing a rise in the burden of both communicable and non-communicable diseases and may further increase the cost of healthcare on governments [14]. The majority of communicable and non-communicable diseases (with 80% of global deaths from chronic diseases) are due to poor personal hygiene and environmental conditions that influence a person’s wellbeing [4]. These conditions account for 75% healthcare spending and annually decline economic output in the USA by $260 billion [15]. The Centers for Disease Control affirms the fact that most chronic diseases can be prevented by lifestyle modifications such as diet and exercise [16]. Most chronic diseases can be averted by focusing more on health prevention and promotion interventions alongside curative care [17]. Although several financial strategies such as taxes and increases in premiums may exist geared towards sustaining pro-poor health insurance schemes, preventive healthcare/public health interventions, such as disease control and prevention, and health promotion activities might be vital. However, no review study to date exists in the literature relating to preventive healthcare and the sustainability of pro-poor health financing schemes. Health promotion and disease prevention interventions in communities/households or at the health facility level are crucial to reducing the occurrence of disease risk factors, morbidities/disease burden, and hospital/clinic attendance for healthcare. Hence, implementing health prevention and promotive activities deliberately can lead to an increase in a healthy population, reduce healthcare costs, and sustain pro-poor health financing schemes. For these reasons, this study seeks to synthesize literature and map evidence on the use of health promotion and disease prevention interventions as a strategy to sustaining pro-poor health financing schemes globally using a systematic scoping review. This also anticipates identifying research gaps for future studies to better shape and sustain pro-poor health financing schemes and continual improvement of access to healthcare irrespective of one’s income status.

## Methods

The study aims to systematically search existing literature and describe the evidence on the role of preventive health services as a strategy for the financial sustainability of pro-poor health financing schemes and identify gaps for future research. To achieve this goal, we will conduct a systematic scoping review by following the steps (identifying the research question, identifying relevant studies, study selection, charting the data, and collating, summarizing, and reporting the results) prescribed by Arksey and O’Malley in 2005 [18] incorporating the Levac and colleagues 2010 recommendations [19] and Peters et al. 2015 guidelines [20]. This is because a scoping review study allows searching of existing literature and examining the extent, range, and nature of research activities to identify research gaps and dissemination of the findings [18]. The Preferred Reporting Items for Systematic reviews and Meta-analysis extension for a scoping review (PRISMA-ScR) checklist will be used to guide the reporting of this study [21], but the development of this protocol was guided by PRISMA-P (Supplementary file 1).

### Identifying the research question

The proposed question for this study is: What evidence exists on the use of health promotive and disease prevention interventions as a strategy to sustaining pro-poor health financing schemes globally?

The sub-review questions will be as follows:

1. What health policies/strategies/interventions exist for sustaining pro-poor health financing schemes globally?
2. What evidence exists on the use of disease prevention intervention as a strategy for sustaining pro-poor health financing schemes globally?
3. What evidence exists on the use of health promotion intervention as a strategy for sustaining pro-poor health financing schemes globally?

The Population, Concept, and Context (PCC) mnemonic used to determine the eligibility of the proposed scoping review question is shown in Table 1.

**Table 1:** PCC framework for defining the eligibility of the studies for the primary research question

P: Population	Pro-poor health financing schemes: these include national health insurance schemes, partial health insurance schemes, and free maternal health services
C: Concept	Preventive health service: This includes intervention/measures to decrease the burden of disease and associated risk factors aimed at sustaining pro-poor health insurance schemes Health promotion: This includes a wide range of social and environmental interventions that are designed to benefit and protect individual people’s health and quality of life by addressing and preventing the root causes of ill health, not just focusing on treatment and cure
C: Context	Sustainability in the global context

### Identify relevant studies

A detailed and comprehensive search will be done on various bibliographic databases to obtain all relevant studies on how preventive healthcare averts most diseases and deaths and sustains the health insurance policies. We will search Academic Search Complete, MEDLINE through PubMed, Web of Science, Google Scholar, SCOPUS, CINAHL, and Science Direct from 1 January 2000 to the last search date in 2021 for relevant published studies to answer the research question. We used a combination of the following keywords to search for relevant studies from the databases: preventive healthcare,’ ‘disease prevention,’ ‘health promotion,’ ‘public health intervention,’ ‘ ‘promotive health,’ ‘health insurance,’ ‘insurance scheme,’ ‘healthcare financing,’ ‘healthcare cost,’ and ‘sustainability’. The search strategy for this study will be developed in consultation with a librarian to ensure all potentially relevant articles are captured. Study design limitations will be removed, but the search language will be limited to English during the database search. Boolean terms, “AND” and “OR,” will be applied to separate keywords. We will also include appropriate Medical Subject Heading (MeSH) terms and keywords to identify relevant studies. Also, we will search the reference list of the included articles and relevant websites such as the World Health Organization for eligible evidence sources. Table 2 shows a pilot search conducted in PubMed to illustrate how feasible the proposed scoping review will be conducted.

**Table 2:** Pilot search in PubMed electronic database

Date	Database	Keywords	Search results
04/03/2021	PubMed	("health promotion"[MeSH Terms] OR ("health"[All Fields] AND "promotion"[All Fields]) OR "health promotion"[All Fields]) OR (("disease"[MeSH Terms] OR "disease"[All Fields]) AND ("prevention and control"[Subheading] OR ("prevention"[All Fields] AND "control"[All Fields]) OR "prevention and control"[All Fields] OR "prevention"[All Fields])) OR ("interventions"[All Fields] AND strategy [All Fields]) AND (pro-poor [All Fields] AND ("insurance, health"[MeSH Terms] OR ("insurance"[All Fields] AND "health"[All Fields]) OR "health insurance"[All Fields] OR ("health"[All Fields] AND "insurance"[All Fields])) AND schemes [All Fields])	289

### Eligibility criteria and study selection

The study selection will be guided by the eligibility criteria as specified under the inclusion/exclusion criteria to ensure that relevant studies are selected.

#### Inclusion criteria

We will include studies that meet the following criteria:

- Articles on pro-poor health financing schemes
- Articles on sustainability of pro-poor health insurance schemes
- Articles presenting findings on health policies/strategies/intervention relating to sustainability of pro-poor health financing schemes
- Articles presenting findings on disease prevention interventions and sustainability of pro-poor health financing schemes
- Articles presenting findings on health promotion interventions and sustainability of pro-poor health financing schemes
- Articles reporting findings on preventive health services and reduction of healthcare cost
- Articles presenting evidence on the relationship between health prevention/promotion and sustainability of pro-poor health financing schemes
- Quantitative, qualitative, and mixed methods study designs
- Articles published in English
- Articles published between 2000 and 2021

#### Exclusion criteria

This proposed study will exclude the following:

- Articles reporting evidence on financial policies for sustaining pro-poor health financing schemes such as increasing subscribers’ premium/taxes
- Articles presenting on stakeholders’ perspective of pro-poor health finding scheme
- Review studies
- Papers published in other languages such as French, Portuguese, Spanish, and Arabic

### Study selection

**Fig. 1:**
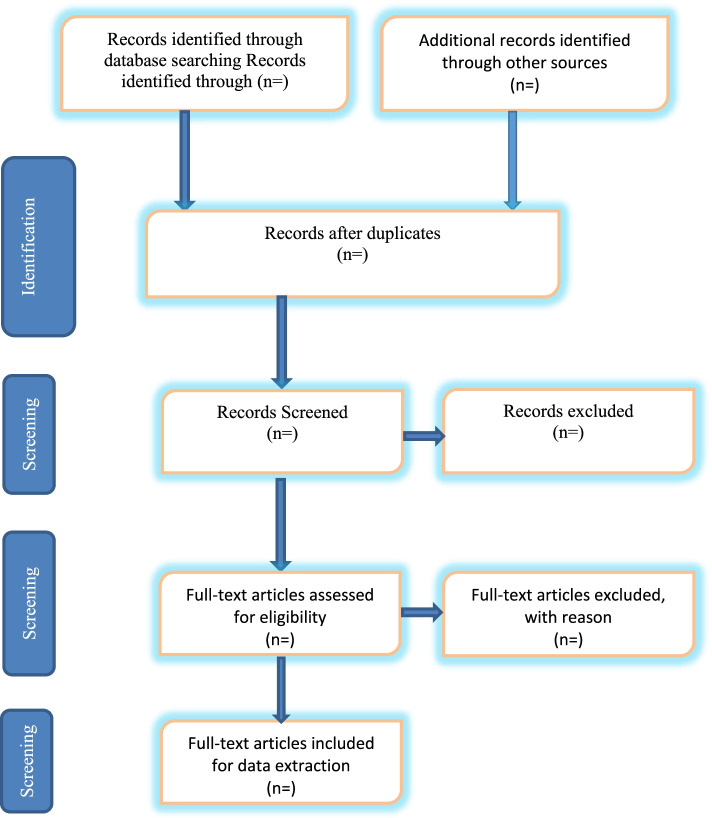
PRISMA 2009 flow diagram

The study selection for this scoping review will involve two stages: title and abstract, and full-text screening. Following the database searches and removal of duplicates, the endnote library created for the study will be shared with the review team. The titles and abstract screening as well as the full-text screening will be done by two independent reviewers. The two independent reviewers will use this study’s eligibility criteria to sort studies into the “inclusive” and “exclusive” criteria. Reviewers’ disparities at the titles and abstract screening stage as well as at the full-text screening stage will be resolved by involving a third reviewer. We will adopt the Preferred Reporting Items for Systematic Reviews and Meta-Analysis (PRISMA) flow diagram to present the screening results of this proposed scoping review (Fig. 1).

### Charting the data

The data from the included studies will be extracted using a hybrid technique that includes both inductive and deductive approaches. We will develop a data charting form for the extraction of relevant data from the included studies. This form will include the following: author and date, study title, objective/aim of the study, study design, study setting, type of pro-poor health financing scheme, health prevention/promotion policy/strategy/intervention, and study finding relating to health promotion and disease prevention intervention strategies, as well as sustainability of pro-poor health financing schemes. The data extraction form will be piloted prior to its usage by two investigators independently, and all necessary amendments made to ensure that it captures all relevant data needed to address the research question, as well as ensure trustworthiness of this study’s findings.

### Collating, summarizing, and reporting the results

Based on initial coding and categorization, thematic content analysis will be employed to define the themes linked with health promotion and disease prevention intervention strategies and sustainability of pro-poor health financing schemes. The themes and sub-themes will be collated, and a narrative summary of the findings relating to health promotion and disease prevention intervention strategies and sustainability of pro-poor health financing schemes reported. A descriptive analysis of the characterictics of the included studies such as the study design, study setting (facility or community-based), study population, and geographic location of study will be conducted using Microsoft Excel, and the frequencies and percentages of each reported.

### Quality appraisal

The 2018 version of the mixed method quality appraisal tool (MMAT) [22] will be used to evaluate the methodological quality of all the included articles using the appropriate section for each study design. The relevance of the study, study design, adequacy and methodology, data collection, analysis of data, and results reported will be examined using the MMAT tool. We will score the quality of each study and the results interpreted as follows: from ≤50% as low quality, 51–75% average quality, and 76–100% high quality. The quality appraisal will be performed by two investigators independently, and any discrepancies between them resolve by a third investigator.

## Discussion

The objective of this scoping review is to explore and describe the evidence on preventive health services as a strategy for sustaining pro-poor health financing schemes globally. Currently, it is proven that the cost of curative medical care is escalating in all countries; hence, disease prevention has gained much attention [23] in order to reduce healthcare costs. We will collate data on health policies/strategies/interventions aiming to help sustain and improve the implementation of pro-poor health financing schemes around the globe. This study’s date limitation (2010–2021) is to enable us to describe the range of research evidence within the last 10 years. We hope to provide evidence of diverse health prevention and promotion policies/strategies used by countries to sustain their pro-poor health financing schemes for possible adoption by other countries. We also anticipate finding research gaps for further studies to help find innovative contextualized health prevention and promotion strategies to sustaining pro-poor health financing schemes especially those in LMICs. The findings will be comprehensively discussed and disseminated at conferences and publication in a peer-reviewed journal.

## Conclusion

Prevention is better than cure. It is anticipated that the outcome of this systematic scoping review research will inform policy-makers, financiers, and implementers of pro-poor health financing schemes on adopting health promotion and disease prevention policies and strategies to sustain pro-poor health financing schemes. Also, evidence provided by this study will help to identify research gaps for future studies such as systematic reviews, meta-analysis, and primary studies to improve health financing schemes through health promotion and preventive healthcare.

## Supplementary Information

**Additional file 1.** PRISMA-P checklist.

## Data Availability

We have duly cited all study and data is presented in the form of references.

## Abbreviations

CDC: Centers for Disease Control
MMAT: Mixed method quality appraisal tool
UHC: Universal Health Coverage
WHO: World Health Organization
PRISMA: Preferred Reporting Items for Systematic Review and Meta-Analysis
SDGs: Sustainable development goals
UN: United Nations
FCTC: Framework Convention on Tobacco Control
LMICs: Low-and-middle-come countries
